# Antenatal care coverage in a low-resource setting: Estimations from the Birhan Cohort

**DOI:** 10.1371/journal.pgph.0001912

**Published:** 2023-11-15

**Authors:** Clara Pons-Duran, Delayehu Bekele, Sebastien Haneuse, Bezawit Mesfin Hunegnaw, Kassahun Alemu, Munir Kassa, Yifru Berhan, Frederick G. B. Goddard, Lisanu Taddesse, Grace J. Chan

**Affiliations:** 1 Department of Epidemiology, Harvard T. H. Chan School of Public Health, Boston, Massachusetts, United States of America; 2 Department of Obstetrics and Gynecology, St. Paul’s Hospital Millennium Medical College, Addis Ababa, Ethiopia; 3 Department of Biostatistics, Harvard T. H. Chan School of Public Health, Boston, Massachusetts, United States of America; 4 Department of Pediatrics and Child Health, St. Paul’s Hospital Millennium Medical College, Addis Ababa, Ethiopia; 5 HaSET Maternal and Child Health Research Program, Addis Ababa, Ethiopia; 6 Department of Epidemiology and Biostatistics, Institute of Public Health, College of Medicine and Health Sciences, University of Gondar, Gondar, Ethiopia; 7 Ministry of Health, Addis Ababa, Ethiopia; 8 St. Paul’s Hospital Millennium Medical College, Addis Ababa, Ethiopia; 9 Department of Pediatrics, Boston Children’s Hospital, Harvard Medical School, Boston, Massachusetts, United States of America; University of Washington, UNITED STATES

## Abstract

Antenatal care (ANC) coverage estimates commonly rely on self-reported data, which may carry biases. Leveraging prospectively collected longitudinal data from the Birhan field site and its pregnancy and birth cohort, the Birhan Cohort, this study aimed to estimate the coverage of ANC, minimizing assumptions and biases due to self-reported information and describing retention patterns in ANC in rural Amhara, Ethiopia. The study population were women enrolled and followed during pregnancy between December 2018 and April 2020. ANC visits were measured by prospective facility chart abstraction and self-report at enrollment. The primary study outcomes were the total number of ANC visits attended during pregnancy and the coverage of at least one, four, or eight ANC visits. Additionally, we estimated ANC retention patterns. We included 2069 women, of which 150 (7.2%) women enrolled <13 weeks of gestation with complete prospective facility reporting. Among these 150 women, ANC coverage of at least one visit was 97.3%, whereas coverage of four visits or more was 34.0%. Among all women, coverage of one ANC visit was 92.3%, while coverage of four or more visits was 28.8%. No women were found to have attended eight or more ANC visits. On retention in care, 70.3% of participants who had an ANC visit between weeks 28 and <36 of gestation did not return for a subsequent visit. Despite the high proportion of pregnant women who accessed ANC at least once in our study area, the coverage of four visits remains low. Further efforts are needed to enhance access to more ANC visits, retain women in care, and adhere to the most recent Ethiopian National ANC guideline of at least eight ANC visits. It is essential to identify the factors that lead a large proportion of women to discontinue ANC follow-up.

## Introduction

Antenatal care (ANC) coverage is an indicator of utilization of essential health services for pregnant women, defined as the proportion of women with a birth in a certain period who attended a specific number of ANC visits for their most recent pregnancy [1, 2]. ANC provides an opportunity to anticipate complications, deliver preventive strategies, and ultimately identify population groups at high risk of experiencing adverse events during pregnancy to prevent them [1]. ANC could contribute to reducing maternal and neonatal mortality figures and improve other maternal and newborn health outcomes [3, 4]. Ethiopia is the second most populous country in Africa, with the fastest growing economy on the continent [5]. Despite an increasing trend in ANC coverage over the past two decades [6], the coverage of at least one ANC visit during pregnancy was lower than 75%, and the proportion of women who remain in care for up to four or more visits was 43% in 2019 [7]. These country estimates are far from the national target of achieving 81% coverage of four or more ANC visits by 2025 [8]. Recently in early 2022, the National ANC guideline was updated, and now a minimum of eight ANC visits during pregnancy is recommended [9].

There is evidence suggesting that self-reports of ANC use are prone to biases. When actual ANC coverage is low, coverage estimates based on self-reports have low specificity [10]. This may be due to long recall periods, social desirability bias, or poor understanding of what constitutes ANC. In Ethiopia and other low-resource settings, there is a lack of studies that validate self-reported data about ANC use, and the reliability of those estimates is unknown. Further, most studies on ANC coverage conducted in Ethiopia are cross-sectional [11–16], and to our knowledge, there is no evidence of ANC estimates based on longitudinal prospective data or direct observation. In addition to the potential biases that self-reported data may imply, cross-sectional studies make it difficult to investigate retention in care patterns and timing of the recommended ANC visits.

Quality and accurate estimates of ANC coverage from different regions in Ethiopia are fundamental to understanding the level of ANC use at local level and to tracking implementation progress of the recently launched National ANC guideline [9]. Reliable estimates are needed to inform the design and delivery of interventions to enhance ANC attendance. This study presents an opportunity to leverage prospectively collected longitudinal data from a maternal and child cohort. We aimed to estimate the coverage of ANC using an approach that minimizes assumptions and biases from self-reported information and to describe retention patterns in ANC in rural Amhara, Ethiopia.

## Methods

### Study design and setting

We conducted a cohort study in the Birhan field site, including 16 villages in Amhara Region, Ethiopia, covering a mid-year population of 77,766, to estimate morbidity and mortality outcomes among 17,108 women of reproductive age and 8,554 children under-five with house-to-house surveillance every three months. The field site is a platform for community and facility-based research and training that was established in 2018, with a focus on maternal and child health [17]. The site includes all health facilities in the catchment area—five public health centers, two public hospitals, and a private hospital. Nested in the site is an open pregnancy and birth cohort, the Birhan Cohort, that enrolls approximately 2,000 pregnant women and their newborns per year with rigorous longitudinal follow-up over the first two years of life and household data linked with health facility information [18]. The catchment area is rural and semi-urban, covers both highland and lowland areas, and includes two different districts, Angolela Tera, and Kewet/Shewa Robit.

We used data from the Birhan Health and Demographic Surveillance System (HDSS) and Birhan Cohort to estimate coverage of ANC-related indicators [17, 18]. The HDSS provides estimates and trends of health and demographic outcomes including morbidity among women of reproductive age and children under two years and births, deaths, marriages, migration in the entire population, and an active pregnancy screening every three months [17]. The pregnancy and birth cohort, generates evidence on pregnancy, birth, and child outcomes using clinical and epidemiological data at both the community and health facility level [18].

### Study population

We used data of women enrolled and followed until delivery in the Birhan Cohort between December 2018 and April 2020. Women were enrolled in the cohort if they had a confirmed pregnancy through pregnancy surveillance as part of the HDSS and provided informed consent. We excluded participants with miscarriages (pregnancy losses <28 weeks) and implausible documented gestational ages at enrollment (≤0 weeks or ≥46 weeks) and/or delivery (<28 weeks or ≥46 weeks). Gestational age was estimated following a hierarchy adapted from the American College of Obstetricians and Gynecologists guidelines [19] and the Global Library of Women’s Medicine [20], using the best available method from ultrasound measurements, date of last menstrual period, fundal height, and maternal recall of gestational age in months. Specifically, ultrasound measurement was used if an ultrasound was done <16 weeks gestation; if ultrasound was only available after 16 weeks, then self-reported last menstrual period was used as long as there was only a limited discrepancy between the last menstrual period and the ultrasound estimates. If ultrasound estimates were not available at all, last menstrual period was used. If neither ultrasound or last menstrual period were available, then fundal height was used if available, or a maternal estimate reported in months if that was the only source of gestational age information [21].

### Study outcomes

Data on ANC visits prior to enrollment were measured by self-report at enrollment. Data on ANC visits after enrollment were collected from facility charts by data collectors immediately after an ANC visit. In a subgroup of women, retrospective facility chart abstraction was done. We defined ANC coverage as the proportion of women enrolled in the Birhan Cohort who attended ANC visits during pregnancy. The primary study outcomes were the total number of ANC visits attended during pregnancy and the coverage of at least one, four, and eight ANC visits.

Secondary study outcomes included retention in care, defined as the continued engagement in facility ANC. We defined those secondary outcomes as the proportion of study participants in care at different gestational age times (ANC 1 window, <16 weeks; ANC 2 window, 16–<28 weeks; ANC 3 window, 28–<36 weeks; and ANC 4 window, ≥36 weeks) and the proportion of participants who were retained in ANC after each of those time windows.

### Analysis

To estimate ANC coverage, we combined the number of visits that were prospectively recorded from enrollment with the number of self-reported visits at the time of enrollment. Since study participants were enrolled at different times during pregnancy, we estimated ANC coverage among the cohort of women who enrolled <13 weeks where most of the ANC visits were recorded prospectively to minimize recall bias. In addition, we also estimated ANC coverage for the entire population. For coverage outcomes, descriptive statistics were undertaken; frequencies, proportions, and Agresti-Coull 95% confidence intervals (CI) [22] were reported for dichotomous outcomes, while median and interquartile range (IQR) were used to report continuous outcomes.

We assessed the quality and reliability of self-reports by comparing the counts of self-reported visits at enrollment and the retrospectively collected visits from charts for a subset of participants. More details of this analysis can be found in the S1 File.

To assess comparability between the study subsample (women enrolled <13 weeks) and the remaining cohort participants, we compared the socio-demographic characteristics and obstetric history of participants enrolled <13 weeks and ≥13 weeks of gestation using descriptive statistics and chi-squared tests, t-test, and Fisher exact tests. Further, women enrolled in the cohort at different gestation age weeks were compared in terms of access to ANC services during second and third trimesters using frequencies and proportions of attendance to at least one ANC visit at different time windows.

To investigate secondary study outcomes, the proportion of participants in care during the window times for the different ANC visits was represented in an alluvial plot. Proportions of women in care during ANC 1 to ANC 4 window times were estimated for the cohort of women enrolled <13 weeks. To our knowledge there are no definitions of ANC retention, or continued attendance in subsequent ANC visits. We defined ANC retention as sequential retention and cumulative retention. Sequential retention is a measure of participants who return to ANC at any point during pregnancy. Sequential retention was calculated for visit X (i.e., ANC 1, ANC 2, ANC 3) as the proportion of participants who returned to visit Y (subsequent visit after visit X) over the total number of women who received ANC visit X as a denominator. For example, sequential retention after ANC 2 would be the proportion of participants who returned to ANC 3 and/or 4 out of the total number who received an ANC 2 visit. Women who attended ANC 2 but missed ANC 3 and then returned for ANC 4 were counted as retained after ANC 2. Cumulative retention was calculated for visit X (i.e. ANC1, ANC2, ANC3 and ANC4) as the proportion of participants who attended visit X and prior ANC visits. For example, women who attended ANC 1 before week 16, ANC 2 between weeks 16 and 28, and ANC 3 between weeks 28 and 36 were considered retained up to ANC 3.

To investigate the potential for selection bias due to loss to follow-up, we conducted a sensitivity analysis estimating coverage of ANC in the missing data under several scenarios each of which assume that ANC coverage among lost women would be lower than that of women followed until delivery. Specifically, for the outcome ‘at least one ANC visit’, we considered the scenario where all women who did not have a visit before being lost never had any ANC visits. For the outcome ‘four ANC visits or more’, we considered three scenarios where women lost to follow-up had ANC coverage in a range from 0% to 90% of the ANC coverage estimated among women who were not lost to follow-up based on the number of visits attended when they were lost. More details of these scenarios can be found in the S2 File.

Analysis was conducted using R version 4.2.2 [23], and Stata version 17 [24].

#### Ethical considerations

Ethical clearance was obtained from the Ethics Review Board (IRB) of Saint Paul’s Hospital Millennium Medical college, (Addis Ababa, Ethiopia) [PM23/274], Boston Children’s Hospital (Boston, United States) [IRB-P00028224], and Harvard T.H. Chan School of Public Health (Boston, United states) [IRB19-0991]. Signed informed consent was obtained from all participants. Additional information regarding the ethical, cultural, and scientific considerations specific to inclusivity in global research is included in the Supporting Information (S1 Checklist).

Authors had no access to participants’ identifiable information during or after data collection unless they were involved in field data collection activities and data quality assurance. All study procedures were followed per protocol and participants confidentiality and anonymity was ensured.

## Results

A total of 2303 women were enrolled in the Birhan Cohort and delivered during the time frame of the study. We excluded 95 (4.1%) women due to miscarriages (62, 2.7%) and implausible gestational ages (33, 1.4%). Among the remaining 2208 women, 139 (6.3%) were lost to follow-up; 2069 women were included in the analysis, of which 150 (7.2%) were enrolled in the cohort before 13 weeks of gestation. The participant characteristics for the groups of women enrolled <13 weeks and ≥13 weeks of gestation were similar (Table 1). There were no statistically significant differences observed for age, residency, socio-economics, obstetric history, and timing of ANC visits (see S3 File). We observed a higher proportion of women with previous history of stillbirth among the participants enrolled in the first trimester. However, the difference in proportion compared to women enrolled later was not statistically significant.

**Table 1:** Characteristics of the study population and by gestational age at enrollment.

**Variables**	**Enrolled <13 weeks (N = 150)**		**Enrolled ≥13 weeks (N = 1919)**		**p-value**	**Total (N = 2069)**	
	**N**	**mean (SD)**	**N**	**mean (SD)**		**N**	**mean (SD)**
Age at conception (years)	150	27.9 (6.4)	1919	27.0 (6.1)	0.12	2069	27.1 (6.1)
Walking time to nearest health facility (minutes)	150	66.2 (57.6)	1909	68.1 (54.7)	0.69	2059	68.0 (54.9)
	**N**	**n (%)**	**N**	**n (%)**	**p-value**	**N**	**n (%)**
Woreda	150		1915		0.44	2065	
Angolela Tera		62 (41.3)		861 (45.0)			923 (44.7)
Kewet		88 (58.7)		1054 (55.0)			1142 (55.3)
Cannot read and write	150	72 (48.0)	1914	866 (45.2)	0.57	2064	938 (45.4)
Wealth index	150		1919		0.38	2069	
1^st^ quintile (the wealthiest)		36 (24.0)		391 (20.4)			427 (20.6)
2^nd^ quintile		28 (18.7)		447 (23.3)			475 (23.0)
3^rd^ quintile		33 (22.0)		386 (20.1)			419 (20.3)
4^th^ quintile		30 (20.0)		328 (17.1)			358 (17.3)
5^th^ quintile (the least wealthy)		23 (15.3)		367 (19.1)			390 (18.8)
Ethnicity	150		1915		1	2065	
Amhara		138 (92.0)		1748 (91.3)			1886 (91.3)
Oromo		8 (5.3)		113 (5.9)			121 (5.9)
Other		4 (2.7)		54 (2.8)			58 (2.8)
Primiparous	150	42 (28.0)	1919	605 (31.5)	0.42	2069	647 (31.3)
History of stillbirth	150	13 (8.7)	1919	97 (5.1)	0.09	2069	110 (5.3)
History of preterm birth	150	6 (4.0)	1919	36 (1.9)	0.12	2069	42 (2.0)

Note: SD—standard deviation

**Fig 1:**
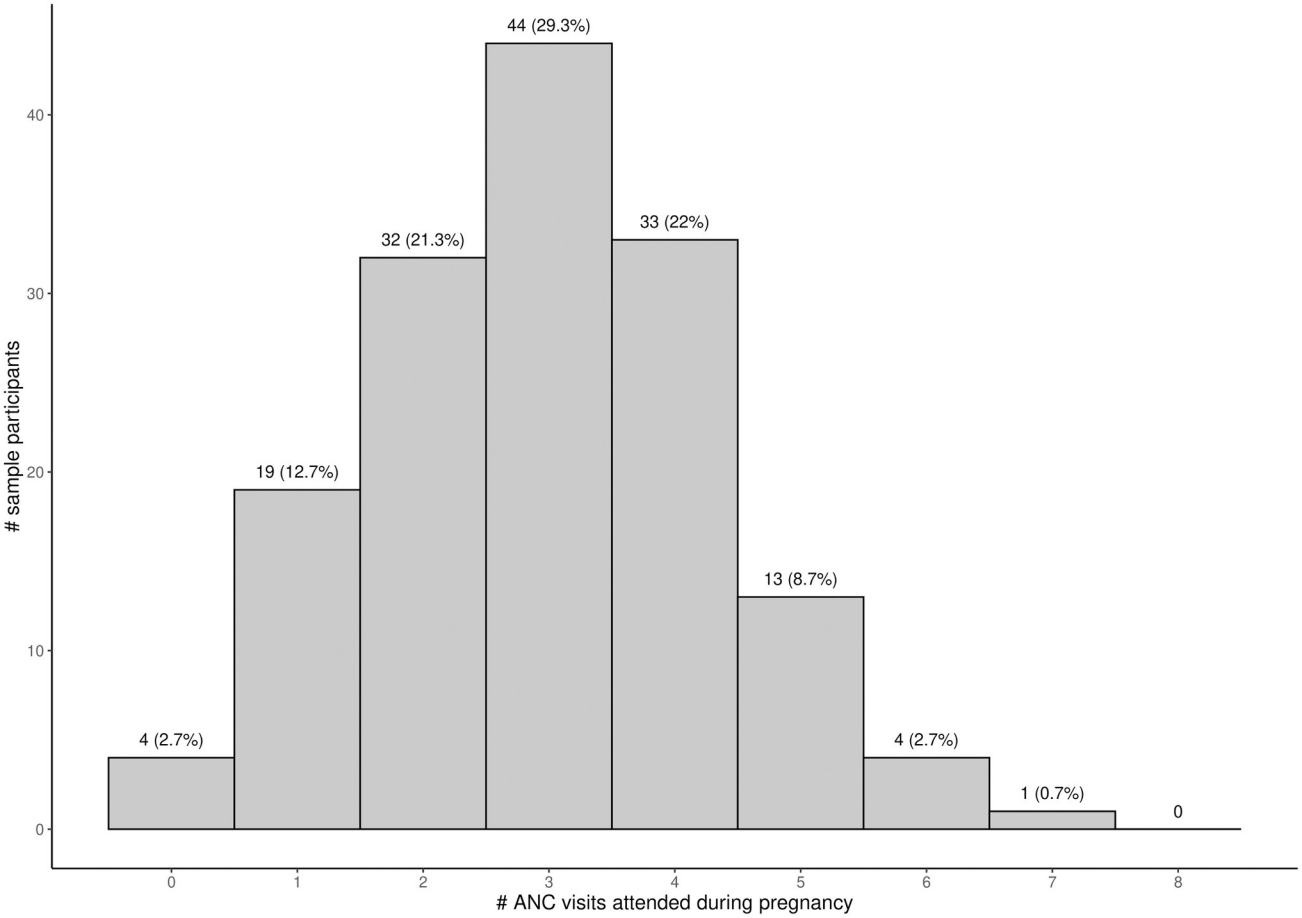
Distribution of ANC visits attended during pregnancy by women enrolled <13 weeks of gestation. Footnote: ANC—antenatal care.

Among the 150 women who enrolled in the cohort before 13 weeks of gestation, 97.3% (95%CI 93.1%–99.2%) had at least one ANC visit, whereas only 34.0% (95%CI 26.9%–41.9%) of the women had four or more ANC visits (Table 2). No women attended eight or more ANC visits. Almost a third of women (44, 29.3%) attended exactly three visits (Fig 1).

**Table 2:** ANC coverage outcomes of women enrolled <13 weeks of gestation.

**ANC outcomes**	**N**	**Median**	**IQR**
Number of ANC visits	150	3	2–4
	**N**	**n**	**% (95% CI)**
At least one ANC visit	150	146	97.3 (93.1–99.2)
Four or more ANC visits	150	51	34.0 (26.9–41.9)
Eight or more ANC visits	150	0	0

Note: ANC—antenatal care; CI—confidence interval; IQR—interquartile range

In the sensitivity analysis, which estimated ANC coverage under several scenarios for the missing data, there was minimal change in the primary outcomes. For example, attendance of at least one ANC visit was 96.4% (95%CI 92.2–98.5) when assuming all women who did not have a visit before being lost to follow-up never accessed ANC. Coverage of four or more ANC visits ranged between 31.4% (95% CI 24.9–38.8%) and 32.9% (95%CI 26.2%–40.3%) when assuming different ranges of ANC coverage among women who were lost to follow-up. Additional details can be found in the S2 File.

Among the total population of 2069 women who enrolled at any time during pregnancy, coverage of at least one and four visits was lower (92.3% and 28.8% respectively), and the proportion of individuals who did not attend any ANC visit during pregnancy was higher (7.7%) than for the subsample of women enrolled <13 weeks (S4 File). There was an agreement close to 50% between the number of self-reported visits at enrollment by study participants and the number of visits recorded in charts, suggesting recall bias among self-reported visits, which were predominantly the source of data for retrospective visits for the women who enrolled later in pregnancy (S1 File). This potential recall bias was observed in both directions, indicating both overreporting and underreporting of actual visits in different cases.

**Fig 2:**
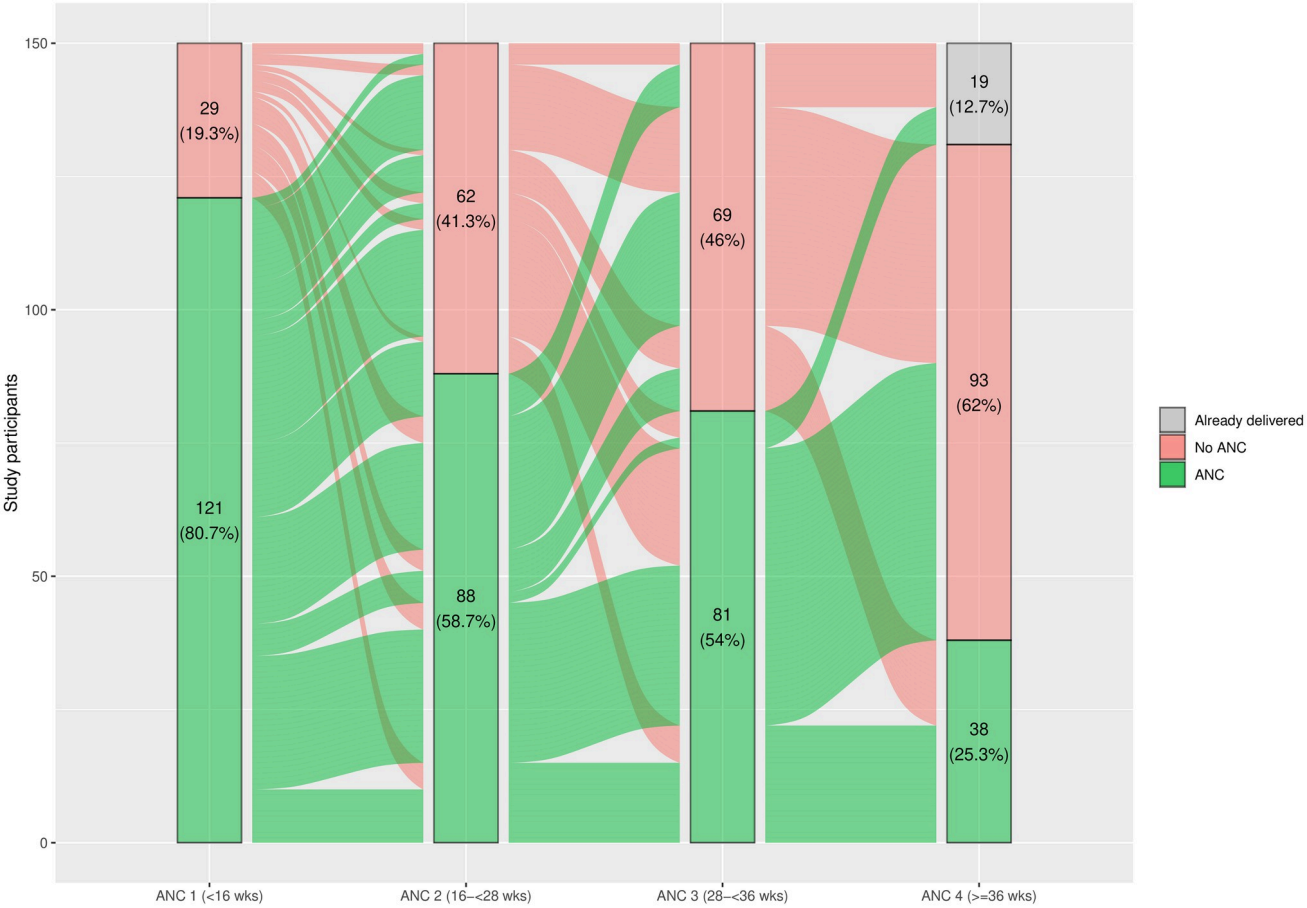
Retention in ANC: Distribution of ANC visits by window time among women enrolled <13 weeks of gestation. Footnote: ANC—antenatal care.

Fig 2 shows that the proportion of women attending ANC visits decreased from 80.7% with an ANC visit before week 16 of gestation to 29.0% with an ANC visit after 36 weeks. Retention in care, both sequential and cumulative, was low. Sequential retention decreased from 86.8% of women who were retained after ANC 1 for subsequent ANC visits, to only 29.7% of women who attended ANC 3 and returned for ANC 4 (Table 3A). Cumulative retention sharply decreased from ANC 1 to ANC 4; only 7.6% of women were retained in all four ANC visits in the appropriate timing (Table 3B).

**Table 3:** ANC retention among women enrolled <13 weeks of gestation.

**A. Sequential retention after each ANC visit regardless of timing of subsequent visits**			
**ANC retention**	**N**	**n**	**% (95% CI)**
Retention after ANC 1 (<16 weeks)	121	105	86.8 (79.5–91.8)
Retention after ANC 2 (16 to <28 weeks)	88	55	62.5 (52.0–71.9)
Retention after ANC 3 (28 to <36 weeks)*	74	22	29.7 (20.5–41.0)
**B. Cumulative retention in ANC visits**			
**ANC retention**	**N**	**n**	**% (95% CI)**
Retention in ANC 1 (<16 weeks)	150	121	80.7 (73.6–86.2)
Retention in ANC 1 and ANC 2 (16 to <28 weeks)	150	69	46.0 (38.2–54.0)
Retention in ANC 1 to ANC 3 (28 to <36 weeks)	150	37	24.7 (18.4–32.2)
Retention in ANC 1 to ANC 4 (≥36 weeks)*	131	10	7.6 (4–13.6)

* The denominator (N) excludes women who delivered before week 36 of gestation

Note: ANC—antenatal care; CI—confidence interval

## Discussion

In this study we found that the coverage of at least one ANC visit during pregnancy is high and close to universal coverage, while coverage of four visits or more remains lower, close to one-third of the sample of women enrolled in the first trimester of pregnancy. No women in our study sample attended eight or more ANC visits. Our findings add valuable information and strengthen the available evidence on ANC coverage that in Ethiopia and other low-resource countries predominantly comes from cross-sectional studies. Longitudinal studies offer more complete sources of information including prospective repeated events, without depending on retrospective self-reported data. We only found cross-sectional studies available in northern Ethiopia to compare with our results. In the Birhan field site between 2018 and 2020, ANC coverage of at least one visit was higher than the results of the 2019 Demographic and Health Survey (DHS) in Amhara region [7, 13]. The Ethiopian mini DHS 2019 reports ANC coverage of pregnancies of the five past years prior to the survey [7]. Thus, it is possible that our coverage of at least one visit is greater due to more attention, policies, and health system resources dedicated to ANC in recent years. On the contrary, our estimates for a minimum of four ANC visits were lower than other studies in Amhara region [7, 13, 15], likely because of the rural nature of the Birhan field site and the potential recall and social desirability bias inherent to the cross-sectional estimates from available studies. No studies reported the coverage of eight or more ANC visits during pregnancy in Ethiopia. Since a minimum of eight ANC visits was not recommended until early 2022, poor coverage of this indicator was expected.

The most common indicator of timely use of ANC services is early ANC attendance, since ANC initiation in the first weeks of pregnancy allows health providers to screen women and conduct tests that are more effective to prevent future complications and assess risks in early weeks [25]. Studies conducted in Ethiopia are not an exception and usually report early ANC attendance as the only indicator of ANC timing [7, 13–15]. However, in order to achieve the recommended number of ANC visits, and to update the information collected in the first visit, timing of subsequent contacts is paramount. To our knowledge, there are no studies on the timing of ANC visits after initiation and on retention in care during pregnancy. Our study demonstrated a trend towards a progressive decrease of ANC attendance during pregnancy, with an especially low proportion of ANC visits after 36 weeks of gestation. The largest drop-out occurred during the window time of the third ANC visit (28 to <36 weeks). The reduced attendance over time may be due to known barriers to ANC utilization that could have a higher impact during the last weeks of pregnancy: negative previous experiences at health facilities, increased fatigue, pregnancy-related signs and symptoms, cultural norms, remoteness that hinders mobility, and lack of family support [6, 26, 27]. In addition, since some ANC components are mainly covered during earlier visits (e.g. complete medical and gynecologic history, ultrasound scan, HIV, hepatitis B and syphilis tests) [9], pregnant women may believe that late visits are less important.

High coverage and continued engagement in ANC are fundamental to reduce preventable pregnancy complications and improve perinatal mortality [3, 4, 28]. In addition, ANC provides the opportunity to be in frequent contact with health providers and improve trust in the health system [29]. ANC attendance is associated with higher rates of facility and skilled-attended delivery [30, 31], which in turn has been observed to decrease perinatal and neonatal mortality compared with home births [32].

Most available ANC coverage estimates from low-resource settings are generated with cross-sectional data and fully based on self-reported information. Our assessment of quality and reliability of self-reported data on ANC visits highlighted a disagreement between data reported by mothers and recorded data in health facility charts, suggesting that self-reports are biased and may decrease the accuracy of estimates. The bias occurred in both directions: in some instances, self-reports overestimated the actual number of visits, while in other cases, there was an underreporting. These results are similar to a validation cross-sectional study conducted in rural China that showed large biases in self-reported data of ANC attendance before 12 weeks of gestation by using the ratio of self-reported over recorded coverage [10]. Our study minimized the potential inaccuracy caused by self-reported information and its inherent biases by leveraging a prospective longitudinal study and restricting the study sample to individuals enrolled early in pregnancy. Our findings flag the possibility that many sources of ANC coverage carry biases and errors. In this context, it is necessary to further explore the use of prospectively collected data and utilize existing pregnancy cohorts to estimate ANC-related outcomes. It is necessary to advocate for increased resource allocation for open cohorts, longitudinal studies, and improved routine information systems and electronic medical records. When longitudinal data sources are not available, new methodological approaches and mitigation strategies are critical to interpreting and using cross-section data for decision making [33].

Study results need to be interpreted in light of some limitations. Attrition, which is inherent to cohort studies, may have introduced selection bias to the results. However, in a sensitivity analysis, study findings remained consistent and similar across three different scenarios with lost to follow-up data. The small sample size of women who enrolled in the cohort before 13 weeks of gestation to estimate ANC coverage and retention indicators led to wide confidence intervals surrounding our estimates. This trade-off was made to minimize biases caused by self-reported information, leading to more accurate estimates. Our results are only generalizable to women with pregnancies that continue beyond 28 weeks of gestation since we excluded women with miscarriages. However, this does not have a big implication for results interpretation since women with miscarriage do not have a complete ANC follow-up due to early end of pregnancy. It was challenging to retrieve data from health facilities outside of the study catchment area, although we suspect the number of women seeking care at facilities outside the catchment were few. While we did not observe any significant differences in baseline characteristics or timing of ANC between women who enrolled <13 weeks of gestation and those enrolled later in pregnancy, it is possible that there exist unmeasured characteristics between the cohort of women who enrolled <13 weeks compared to the entire cohort sample. Finally, data collection took place before the COVID-19 pandemic, between 2018 and 2020. It is possible that ANC coverage may have declined during the pandemic, as indicated by some reports [34] and we assume that ANC coverage has returned to coverage levels similar to pre pandemic [35].

Despite excellent coverage of one or more ANC visits, adequate coverage of the previously recommended four or more ANC visits has not been reached yet in Ethiopia. The recent launch in 2022 of the National ANC guideline to achieve eight or more ANC visits creates an opportunity to continue devoting efforts and resources to increasing adherence and retention of pregnant women in ANC all over the country [9]. As health system and health professionals target adherence to the new ANC guideline, the number of visits attended by pregnant women may potentially experience an increase. As National ANC guidelines aligned with the World Health Organization (WHO) recommendations are being rolled out in Ethiopia and in other low-resource countries, longitudinal studies as well as quality routine information systems and complete electronic medical records provide reliable and accurate metrics for the evaluation and monitoring of implementation of these new guidelines.

## Conclusion

This study presents a rigorous methodological approach to accurately estimate ANC coverage, leveraging longitudinal data from a pregnancy and birth cohort in Amhara region, Ethiopia. Despite the high proportion of pregnant women who accessed ANC at least once in the study area, further efforts are needed to enhance access to more ANC visits and retain women in care during pregnancy to achieve the global WHO target of eight ANC visits [1]. Furthermore, it is essential to identify and mitigate the barriers that contribute to the large proportion of women who discontinue ANC follow-up after initial ANC visits. Longitudinal prospective data collection through cohort studies, routine health information and electronic medical records should be improved and promoted to minimize the general reliance on self-reports of ANC attendance that could lead to biased estimates and to obtain useful metrics of visits timing to describe patterns of retention in care.

## Supporting information

S1 Checklist Inclusivity in global research. (DOCX)

S2 Checklist STROBE checklist. (DOC)

S1 File Quality and reliability assessment of self-reports. It includes: Table A. Distribution of recorded visits that occurred before enrollment and self-reported visits at enrollment for individuals with at least one recorded visit before enrollment.
(DOCX)

S2 File Sensitivity analysis. It includes: Table A. Sensitivity analysis: scenarios of ANC coverage of four or more visits.
(DOCX)

S3 File Comparability assessment. It includes: Table A. Timing of ANC visits across different gestational ages at enrollment.
(DOCX)

S4 File ANC coverage in the entire Birhan Cohort. It includes: Fig A. Distribution of ANC visits attended during pregnancy by women enrolled in the Birhan Cohort; Table A. ANC coverage outcomes of women enrolled in the Birhan Cohort.
(DOCX)
