# Supplementary material for: Antenatal care coverage in a low-resource setting: Estimations from the Birhan Cohort
Source: PLOS Glob Public Health. 2023 Nov 15;3(11):e0001912. doi: 10.1371/journal.pgph.0001912 (PMC10651002; doi:10.1371/journal.pgph.0001912)
Supplement: S1 File — It includes: Table A. Distribution of recorded visits that occurred before enrollment and self-reported visits at enrollment for individuals with at least one recorded visit before enrollment. (DOCX) [file pgph.0001912.s003.docx]

**S1 File**

**Quality and reliability assessment of self-reports**

Methods

Retrospective facility chart abstraction was conducted for a subset of cohort participants. In order to assess the quality and reliability of self-reports, which were the main source of information for the ANC coverage analysis of the entire sample of 2069 pregnant women, we compared the counts of self-reported visits at enrollment and the retrospectively collected visits from charts for the subset of participants that had at least one retrospectively abstracted visit that took place before enrollment. The counts of visits were described using frequencies and percentages.

Results

The quality and reliability assessment of self-reports was performed among 241 women who had at least one visit that occurred before enrollment collected from facility charts, regardless of their date of delivery or having been lost to follow-up.

Table A shows poor agreement between the number of self-reported visits at enrollment by study participants, and the number of visits recorded in charts that occurred before enrollment. The shaded cells indicate the proportion of cases with agreement among both counts, generally being close to 50%, and being highest for the counts of three visits (68.8%).

*Table A. Distribution of recorded visits that occurred before enrollment and self-reported visits at enrollment for individuals with at least one recorded visit before enrollment (N=241)*

| **Recorded visits that occurred before enrollment** | **N** | **Self-reported visits** | | | | | | | |
| --- | --- | --- | --- | --- | --- | --- | --- | --- | --- |
|  |  | **0** | **1** | **2** | **3** | **4** | **5** | **6** | **7** |
| 1 | 165 | 20.0% | **41.2%** | 27.3% | 8.5% | 3.0% | 0 | 0 | 0 |
| 2 | 53 | 5.7% | 3.8% | **50.9%** | 26.4% | 7.5% | 0 | 3.8% | 1.9% |
| 3 | 16 | 12.5% | 6.3% | 0 | **68.8%** | 12.5% | 0 | 0 | 0 |
| 4 | 7 | 14.3% | 0 | 0 | 28.6% | **57.1%** | 0 | 0 | 0 |
