# Supplementary material for: Antenatal care coverage in a low-resource setting: Estimations from the Birhan Cohort
Source: PLOS Glob Public Health. 2023 Nov 15;3(11):e0001912. doi: 10.1371/journal.pgph.0001912 (PMC10651002; doi:10.1371/journal.pgph.0001912)
Supplement: S2 File — It includes: Table A. Sensitivity analysis: scenarios of ANC coverage of four or more visits. (DOCX) [file pgph.0001912.s004.docx]

**S2 File**

**Sensitivity analysis**

A sensitivity analysis was conducted with the 17 women who were lost to follow-up and excluded from the analysis among those enrolled <13 weeks.

Coverage of at least one ANC visit. Fifteen (88.2%) women had attended at least one ANC visit before being lost. In the most extreme case where the remaining two participants failed to attend any visit, the estimate of attendance to at least one ANC visit would be 96.4% (95%CI 92.2 - 98.5).

Coverage of four or more ANC visits. The number of ANC visits attended by the 17 women who were lost to follow-up before being lost are displayed in the second column of Table A. The three scenarios described in Table A considered different ANC coverage values for those participants, depending on the number of visits attended and the estimated coverage of four or more ANC visits among participants with complete follow-up (34.0%, Table 2 of the article).

*Table A.* *Sensitivity analysis: scenarios of ANC coverage of four or more visits*

| **Visits before LTFU** | **n** | **Scenario 1** | **Scenario 2** | | **Scenario 3** |
| --- | --- | --- | --- | --- | --- |
| 0 | 2 | **0%** of 34%* | **10%** of 34% | | **25%** of 34% |
| 1 | 4 | **10%** of 34% | **25%** of 34% | | **50%** of 34% |
| 2 | 6 | **25%** of 34% | **50%** of 34% | | **75%** of 34% |
| 3 | 5 | **50%** of 34% | **75%** of 34% | | **90%** of 34% |
| Total LTFU | 17 |  | |  |  |
| ANC coverage of four or more visits^#^ |  | 31.4% (24.9% - 38.8%) | | 32.2% (25.5% - 39.6%) | 32.9% (26.2% - 40.3%) |

**34% is the estimated coverage of four or more ANC visits among women with complete follow-up.*

*^#^The denominator of the ANC coverage of four or more visits estimates of the sensitivity analysis is 167: 150 participants with complete follow-up plus 17 lost participants.*

*Note: ANC – antenatal care, LTFU – lost to follow-up*
