# Supplementary material for: Antenatal care coverage in a low-resource setting: Estimations from the Birhan Cohort
Source: PLOS Glob Public Health. 2023 Nov 15;3(11):e0001912. doi: 10.1371/journal.pgph.0001912 (PMC10651002; doi:10.1371/journal.pgph.0001912)
Supplement: S3 File — It includes: Table A. Timing of ANC visits across different gestational ages at enrollment. (DOCX) [file pgph.0001912.s005.docx]

**S3 File**

**Comparability assessment**

Comparability between the study sample (women enrolled <13 weeks) and the remaining cohort participants was further assessed in terms of access to ANC services during second and third trimesters.

We observed a similar timing of ANC visits across different gestational ages at enrollment. Women enrolled <13 weeks and those enrolled later showed a similar pattern of ANC use during second and third trimesters of pregnancy. Over 60% of the sample had a visit between weeks 17 and 29 of gestation, half of the participants attended at least one ANC visit between weeks 29 and 36, and a quarter had a visit at or after week 37, regardless of gestational age at enrollment.

*Table A. Timing of ANC visits across different gestational ages at enrollment*

|  |  | **Timing of ANC visits** | | |
| --- | --- | --- | --- | --- |
| **Gestational age at enrollment** | **N** | **≥17 to <29 weeks**  n (%) | **≥ 29 to <37 weeks**  n (%) | **≥37 weeks**  n (%) |
| 0-12 weeks | 150 | 94 (62.7) | 77 (51.3) | 38 (25.3) |
| 13-16 weeks | 168 | 103 (61.3) | 82 (48.8) | 51 (30.4) |
| 17-30 weeks | 268 | 215 (80.2)* | 145 (54.1) | 66 (24.6) |
| 21-24 weeks | 333 | 238 (71.5)* | 163 (48.9) | 78 (23.4) |
| 25-28 weeks | 317 | 185 (58.4)* | 163 (51.4) | 81 (25.6) |
| 29-32 weeks | 271 | - | 172 (63.5)* | 74 (27.3) |
| 33-36 weeks | 562 | - | 84 (14.9)* | 121 (21.5) |

** Proportion of women enrolled in a specific gestational age week that falls within the ANC window period.*

*Note: ANC – antenatal care*
