# Supplementary material for: Antenatal care coverage in a low-resource setting: Estimations from the Birhan Cohort
Source: PLOS Glob Public Health. 2023 Nov 15;3(11):e0001912. doi: 10.1371/journal.pgph.0001912 (PMC10651002; doi:10.1371/journal.pgph.0001912)
Supplement: S4 File — It includes: Fig A. Distribution of ANC visits attended during pregnancy by women enrolled in the Birhan Cohort; Table A. ANC coverage outcomes of women enrolled in the Birhan Cohort. (DOCX) [file pgph.0001912.s006.docx]

**S4 File**

**ANC coverage in the entire Birhan Cohort**

*Fig A. Distribution of ANC visits attended during pregnancy by women enrolled in the Birhan Cohort*


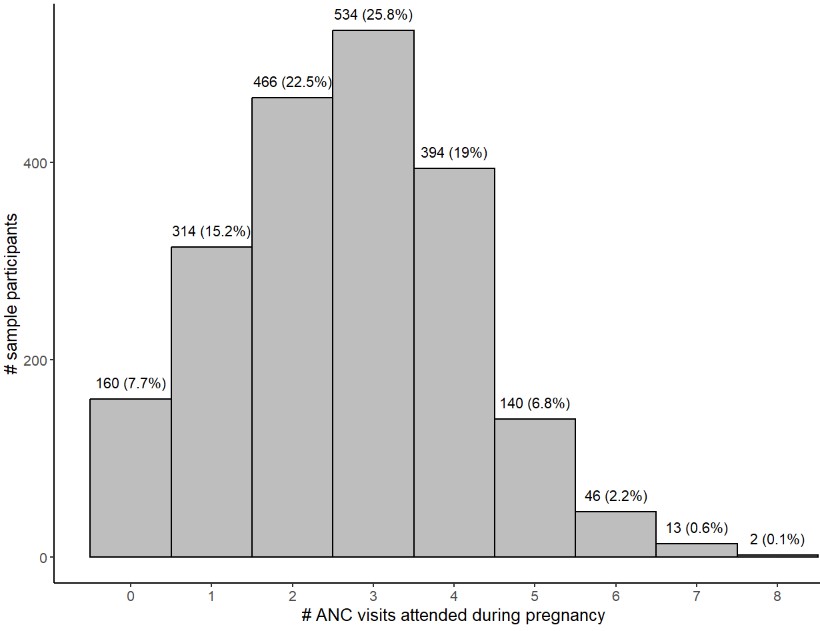


*Table A. ANC coverage outcomes of women enrolled in the Birhan Cohort*

| **ANC outcomes** | **N** | **Median** | **IQR** |
| --- | --- | --- | --- |
| Number of ANC visits | 2069 | 3 | 2-4 |
|  | **N** | **n** | **% (95% CI)** |
| At least one ANC visit | 2069 | 1909 | 92.3 (91.0 – 93.3) |
| Four or more ANC visits | 2069 | 595 | 28.8 (26.8 – 30.7) |
| Eight or more ANC visits | 2069 | 2 | 0.1 (0 – 0.4) |

*Note: ANC – antenatal care; CI – confidence interval; IQR – interquartile range*
